# Evaluation of Volumetric Reference Ranges for SPECT MPI Parameters and the Predictive Power of Dyssynchrony Parameters: A Cross-Sectional Study

**DOI:** 10.2174/0115734056444403260202075613

**Published:** 2026-02-13

**Authors:** Ahmad Alenezi, Zekun Jiang, Mohammad Alshaheen, Zain Khurshid, Qamar Almutawa, Ma’en AlOdat

**Affiliations:** 1 Department of Radiologic Sciences, Kuwait University, Kuwait City, Kuwait; 2 West China Biomedical Big Data Centre, West China Hospital, Sichuan University, Chengdu, China; 3 Department of Nuclear Medicine, Jahra Hospital, Ministry of Health, Kuwait City, Kuwait; 4 Department of Medical Laboratory Science, Faculty of Allied Medical Science, Mutah University, Karak, Jordan

**Keywords:** SPECT, MPI, Cardiac imaging, End-diastolic volume, Myocardial perfusion imaging, Stress

## Abstract

**Introduction:**

This study aims to evaluate reference ranges for SPECT Myocardial Perfusion Imaging (MPI) parameters using Myoview^®^ (tetrofosmin) radiopharmaceutical and Myovation^®^ processing software. This study also aims to provide a reference range for future MPI quantitative studies in patients with suspected heart disease and to identify significant variables associated with an abnormal left ventricular ejection fraction.

**Methods:**

Data were retrospectively collected from 1,100 MPI studies (2017-2024) with 932 participants included after excluding poor-quality images. Imaging was performed using a GE SPECT/CT Optima NM/CT640 camera, and images were reconstructed using the OSEM algorithm (Myovation^®^). Volumetric and quantitative parameters were extracted for analysis (*e.g.*, Left Ventricular Ejection Fraction (LVEF), End-Systolic Volume (ESV), End-Diastolic Volume (EDV), Stroke Volume (SV), and dyssynchrony parameters). Reference ranges were derived using descriptive statistics, and comparative analyses examined how parameters varied by sex and age. Regression analysis and Receiver Operating Characteristic (ROC) curves were used to assess the relationship between abnormal LVEF and dyssynchrony indices.

**Results:**

The study analysed 932 participants under stress and 462 at rest, yielding adequate statistical power. Average LVEF was 68% in both conditions. At stress, mean EDV was 95.1 mL and mean ESV was 34.7 mL; corresponding values at rest were 104.8 mL and 40.1 mL. Diagnosis significantly influenced all volumetric and dyssynchrony parameters at rest and during stress (all *p* < 0.001), showing progressive ventricular dilation, reduced LVEF, and increased dyssynchrony from the normal to the ischemic and infarcted groups. Sex significantly affected LVEF and ventricular volumes, with females exhibiting higher LVEF and smaller volumes, while age had minimal effects. Resting dyssynchrony indices correlated strongly with stress LVEF, particularly in diseased groups. Logistic regression demonstrated good discrimination (AUC = 0.80) and calibration, identifying resting volumetric and clinical factors as independent predictors of abnormal stress LVEF.

**Discussion:**

This study defines sex- and age-specific reference ranges for gated SPECT MPI–derived ventricular function in a Kuwaiti population. Ventricular volumes, systolic function, and dyssynchrony varied significantly by sex and diagnosis, with progressive impairment across disease groups. Logistic regression analysis with multiple variables identified resting volumetric indices and demographic characteristics, rather than dyssynchrony measures, as the primary independent predictors of abnormal left ventricular function during stress. The model demonstrated good discriminatory ability and calibration.

**Conclusion:**

Sex- and age-specific reference ranges for gated SPECT MPI reveal clinically meaningful variation in ventricular function and dyssynchrony by diagnosis. Logistic regression findings indicate that conventional ventricular volumes and patient characteristics primarily drive stress systolic impairment, while dyssynchrony indices offer complementary but not independent prognostic value.

## 1 INTRODUCTION

Quantitative imaging involves the numerical analysis of radiological data to link imaging-derived metrics with clinical findings. This process includes several key steps such as image acquisition, preprocessing, segmentation, and parameter computation, providing detailed insights into tissue characteristics, including shape, motion, and heterogeneity. Quantitative imaging has potential applications in evaluating cardiac abnormalities, including assessment of tissue volume changes [1] and mechanical dyssynchrony [2, 3]. Originally used in oncology to characterize tissue heterogeneity and its association with survival outcomes, quantitative imaging now provides a minimally invasive approach to studying cardiac function and physiology. Unlike coronary angiography, quantitative imaging evaluates the entire myocardial volume, enabling prediction of abnormality severity, treatment response, and potential clinical outcomes [4, 5]. Additionally, its non-invasive nature supports integration of multimodality data, including SPECT, PET, CT, and MRI, with clinical, laboratory, histological, and genomic information through advanced computational techniques and machine learning frameworks.

Despite these advantages, applying quantitative imaging to cardiac studies remains challenging due to the absence of standardized methodological frameworks. For instance, radionuclide angiography-based Left Ventricular Ejection Fraction (LVEF) thresholds differ across guidelines. The European Society of Cardiology defines LVEF below 50% as abnormal [5], while the British Nuclear Medicine Society offers a more detailed classification: mildly impaired (40–50%), moderately impaired (30–40%), and severely impaired (20–30%), with values above 50% considered normal. Similarly, the American Society of Nuclear Medicine and Molecular Imaging considers LVEF ≥50% normal and recommends discontinuing cardiotoxic therapies if LVEF falls below 30% [6]. Although these guidelines reflect clinical variability, they lack quantitative imaging-specific frameworks that include acquisition, preprocessing, segmentation, and standardization steps. This limitation reduces reproducibility and cross-comparison of conventional volumetric and phase-derived parameters such as LVEF, EDV, ESV, and entropy, and the issue extends to SPECT cardiac imaging.

Sex differences in cardiac chamber size and systolic function have been consistently reported in both Western and Asian populations. In a large cohort, Li *et al.* [7] showed that men had significantly larger left-ventricular end-diastolic and end-systolic volumes than women (*p* < 0.001), even after adjusting for body surface area, while women demonstrated higher ejection fractions when measured using gated myocardial perfusion SPECT. A high prevalence of small-heart geometry, particularly among women (> 60%), contributed to these functional differences. The investigators also noted that algorithm-related differences (QGS vs 4D-MSPECT) affected measured ejection fractions, highlighting the impact of both biological and methodological factors. These findings highlight the importance of establishing sex- and population-specific reference ranges for ventricular volumes and ejection fraction, as well as careful consideration of reconstruction algorithms when interpreting myocardial perfusion data [7].

Age has a well-documented influence on left ventricular performance and geometry. Several studies have demonstrated that ejection fraction tends to remain stable or slightly increase with age, particularly in women, while end-diastolic and end-systolic volumes gradually decrease, reflecting ventricular stiffening and reduced compliance [7, 8]. These changes are believed to result from age-associated myocardial remodeling and a decrease in ventricular cavity size, which may artificially elevate LVEF values in older individuals, especially when assessed by gated SPECT imaging. Therefore, establishing age-adjusted normal limits is essential for accurate interpretation of functional cardiac parameters.

Another critical challenge is the lack of agreement in reported dyssynchrony and volumetric values across studies, emphasizing the need for standardized evaluation. Variability in preprocessing and image processing methods can significantly affect results, particularly for parameters like entropy, which quantifies the unpredictability of phase distribution within a Region of Interest (ROI) and may reflect underlying mechanical abnormalities [5, 9]. For example, O'Connell *et al.* [10, 11] showed the potential utility of entropy and synchrony in radionuclide angiography for quantifying ventricular mechanical coordination, though their study did not address acquisition-related variability. Similarly, Jiménez-Ángeles *et al.* [11] emphasized that phase-imaging parameters can vary across acquisition methods, especially in healthy individuals, underscoring the need for standardized imaging pipelines.

While a few studies have explored entropy and synchrony for detecting dyssynchrony in SPECT MPI [12], their findings were limited by small cohorts and reliance on manual or semi-automated segmentation. Most published studies do not describe segmentation algorithms in sufficient detail or assess how variations in acquisition and reconstruction parameters influence the derived volumes and phase metrics, particularly in gated SPECT MPI.

Recognizing the need for standardized quantitative interpretation in cardiac SPECT MPI, this study focuses on establishing robust, sex-specific reference benchmarks for conventional functional and phase-based parameters using the Myoview^®^ radiopharmaceutical. While high-dimensional radiomic texture features were not evaluated, the analysis emphasizes methodological consistency, reproducibility, and clinically interpretable metrics. By defining population-specific reference ranges for LVEF, EDV, ESV, stroke volume, and phase-derived synchrony indices, this work provides a reliable quantitative baseline that may facilitate future methodologically rigorous investigations, including advanced analytical and predictive approaches in cardiac SPECT MPI.

## 2 METHODS

### 2.1 Data Collection

The inclusion criteria for this study included participants aged 18 and older, comprising both healthy individuals and those with diagnosed cardiac conditions, to ensure a representative sample of the population. Exclusion criteria included individuals with incomplete data, severe non-cardiac medical conditions, poor image quality, or different imaging modalities. These criteria were set to maintain the quality and integrity of the study and to ensure the establishment of robust quantitative standards. Participants were divided into three age groups: Senior (> 60 years old), Middle (39-59 years old), and Young (18-38 years old). Using approved clinical reports, we extracted reasons for referral and recoded the Diagnosis and History categories based on disease similarity (Table **1**). This process ensured consistency in categorizing cardiac conditions and improved the comparability of data across different participant groups Figs. (**1** and **2**).

### 2.2 Sex and Gender Considerations

Sex and gender considerations were addressed in accordance with the SAGER guidelines [13]. Participants were stratified by biological sex (male, female) as recorded in clinical documentation. Sex-specific analyses were conducted for all cardiac parameters and dyssynchrony metrics, and sex-disaggregated data are reported throughout the results.

### 2.3 Normal Cohort Definition

Within the retrospective cohort, patients were classified as “normal” if they showed normal myocardial perfusion, normal wall motion, and normal wall thickening on both stress and rest gated SPECT MPI images. Classification was based on clinical reports verified by two board-certified nuclear medicine consultants. Patients in this group had no prior history of coronary artery disease, myocardial infarction, cardiomyopathy, or valvular disorders, and no significant ECG abnormalities at the time of referral. Although all cases were referred for clinical MPI, those meeting these inclusion criteria were considered representative of a physiologically normal or low-risk population. This approach is consistent with previously published methods used to establish reference databases for SPECT MPI [14].

### 2.4 Normal Range

The normal reference group was derived from our patient cohort and included only subjects who demonstrated normal perfusion, wall motion, and wall thickening on gated SPECT MPI, with no prior history of coronary artery disease, revascularization, or ECG abnormalities. Patients with diabetes, hypertension, or other cardiovascular risk factors were excluded. This approach follows established methodologies for developing normal databases for SPECT MPI [15-17]. The mean values of cardiac parameters across all age groups in Diagnosis 1 (the normal heart parameters group) were aggregated and used as the standard reference (Mean ± SD) for comparisons with other diagnostic groups (age groups and disease groups). To establish normal ranges, this part of the study excluded patients with known Coronary Artery Disease (CAD), abnormal perfusion, or significant comorbidities. Only participants with normal clinical reports, unremarkable stress/rest myocardial perfusion imaging, and no history of cardiovascular or systemic conditions were included. This approach ensures that derived normal ranges reflect values from a healthy population, consistent with standard practices for defining normal physiological parameters. These ranges provide a baseline for identifying pathological deviations and establishing reference intervals for quantitative studies.

### 2.5 Image Processing

**Fig (1):**
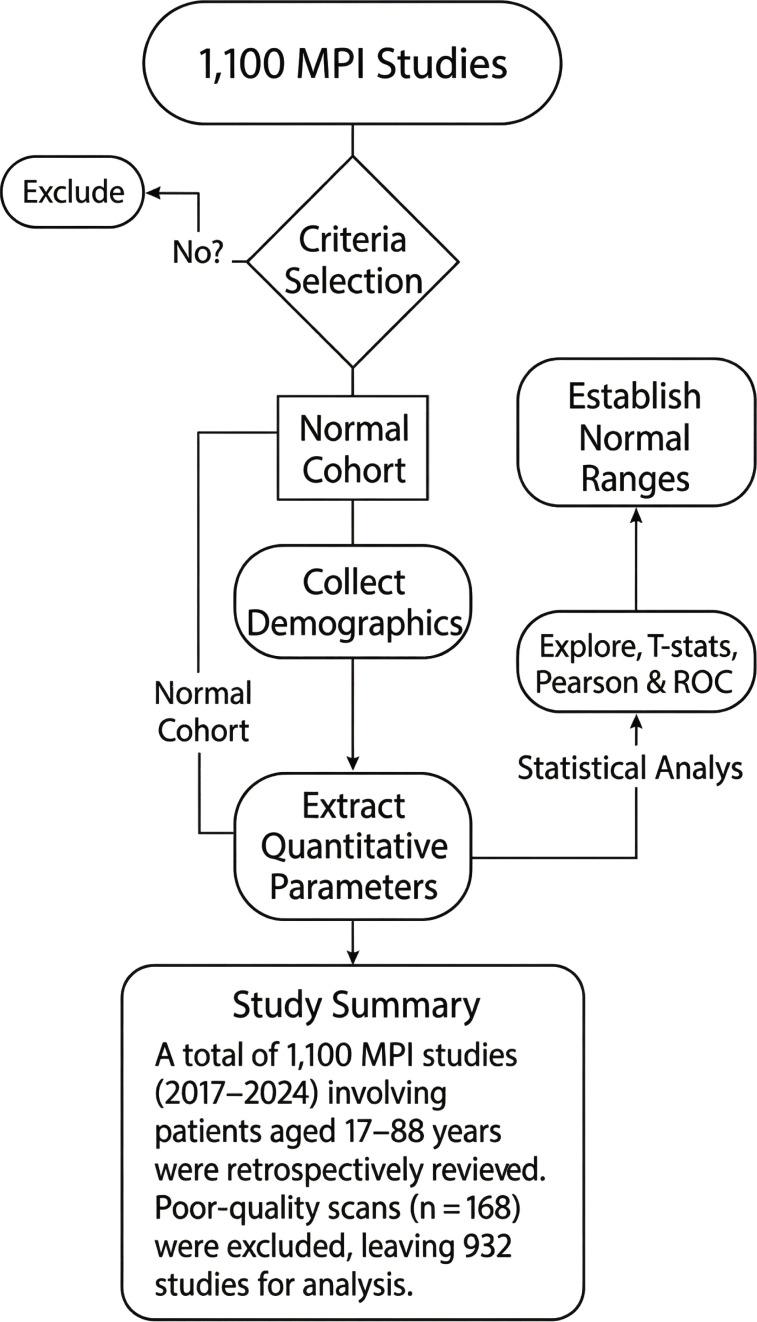
Flowchart summarizing the selection and analysis of myocardial perfusion imaging (MPI) studies. From 1,100 studies (2017–2024), poor-quality scans (n = 168) were excluded, leaving 932 for analysis. Demographics and quantitative parameters were collected from the normal cohort, and statistical tests, including t-tests, Pearson correlations, and ROC analyses, were used to establish normal reference ranges.

A total of 1,100 MPI studies (from 1100 patients) were retrospectively selected between 2017 and 2024, involving patients aged 17 to 88 years. Approximately 168 cases were excluded due to extremely poor image quality (*e.g.*, low target-to-nontarget ratio), leaving 932 cases for analysis (Fig. **1**). All images were acquired in the supine position with 8-frame Electrocardiogram (ECG)-gating and a 2-day rest/stress protocol. Notably, the 2-day protocol is a widely practiced standard in the Arabian Gulf region, though less common in other parts of the world. The imaging procedures were performed using single-photon emission computed tomography/x-ray computed tomography (SPECT/CT) gamma camera equipment, specifically the GE SPECT/CT Optima NM/CT640.

The raw data for analysis were obtained from the Picture Archiving and Communication System (PACS) at the Nuclear Medicine Department of Al-Jahra New Hospital. The radiopharmaceutical used was Technetium 99m tetrofosmin (^99m^Tc-Myoview^®^), administered as a standard dose of 740 MBq (20-30 mCi) adjusted according to weight. All SPECT images were reconstructed using the Ordered-Subset Expectation Maximization (OSEM) algorithm with 2 iterations and 10 subsets. The same filtering technique was applied uniformly to all images using a Butterworth filter of order 5 (power factor = 10 for all critical frequencies) with a cutoff frequency of 0.3. This consistent methodology ensured reproducibility across all imaging data.

### 2.6 Segmentation

Image segmentation was performed using the Myovation^®^ software package (GE Healthcare, Chicago, IL, USA), which applies a semiautomated algorithm to delineate the left ventricular endocardial and epicardial borders based on count density distribution from gated SPECT datasets. All automated contours were visually inspected and, when necessary, manually adjusted by an experienced operator to ensure anatomical accuracy and exclude extracardiac activity. The segmented axes datasets were then exported for further quantitative analysis. Subsequent image processing and computation of volumetric and phase parameters were conducted in MATLAB (MathWorks, Natick, MA, USA) using custom scripts applied to the exported Myovation^®^ data.

### 2.7 Extracted Quantitative Parameters

Gray-scale-based features were extracted to quantify cardiac tissue variations caused by disease. Volume parameters included Left Ventricular Ejection Fraction (LVEF), End Systolic Volume (ESV), End Diastolic Volume (EDV), and Stroke Volume (SV). These parameters were measured for both stress (suffix -S, *e.g.*, LVEF-S) and rest (suffix -R, *e.g.*, LVEF-R) images.

Phase-based features quantified dyssynchrony parameters using phase images to numerically assess the degree of disorder within the Region of Interest (ROI). These features aimed to reflect myocardial tissue characteristics linked to cardiac mechanical dysfunction. Extracted phase-based features included mean phase angles (Mean Angle), standard deviation of phase angles (SD°), and quartiles such as the 25% angle (at 25°), 75% angle (at 75°), and 95% angle (at 95°). The lower and upper limits of angles (LL and UL) were also recorded. Similar to volume parameters, all phase parameters were measured for both stress (-S) and rest (-R) images. All images were processed using the clinical software Myovation^®^ package (GE Healthcare, Chicago, IL, USA) and cross-verified with a novel MATLAB-based parameter extraction code [7, 18]. This dual approach improved accuracy and validated the extracted quantitative and qualitative features for consistency and reproducibility.

### 2.8 Groups Stratification

The study divided participants into groups by sex (male, female) and age category (Young: 18–38, Middle: 39–59, Senior: >60) to examine differences in cardiac parameters and dyssynchrony metrics. Within the overall sample of 932 participants, descriptive and inferential analyses were performed separately for Stress (S) and Rest (R) conditions, allowing direct comparison of rest-stress effects across groups. Sex comparisons used subgroup analyses (*e.g.*, middle-aged males versus middle-aged females) and independent t-tests where appropriate, while age-stratified analyses assessed age-related trends within each sex. Diagnoses were grouped into multiple categories (normal *vs.* abnormal, and additional disease groupings), and analyses were conducted both across the entire cohort and within these stratified partitions. Correlations and regression models were used to explore relationships between anthropometric factors (height, weight), age, and quantitative parameters (LVEF-S, EDV-S, ESV-S, SV-S, Mean Angle-S, SD-S, and their rest equivalents), with significance testing (p-values) and effect sizes reported. The stratification approach was designed to reveal potential heterogeneity in quantitative signatures across sex, age, and clinical diagnosis, while maintaining consistency in imaging protocols and processing across groups.

Clinical reports were grouped into three diagnostic categories based on SPECT MPI interpretations. Group 1 included normal studies with no scintigraphic evidence of ischemia; Group 2 comprised cases showing reversible perfusion defects indicating stress-induced ischemia; and Group 3 included fixed or mixed defects consistent with myocardial infarction or infarct-related ischemia (Table **1**).

### 2.9 Statistical Analysis

Statistical analyses were performed using a general linear model with clinical diagnosis (3 levels), age range (3 levels), and sex (2 levels) entered as fixed between-subjects factors and the resting and stress LV volumetric and dyssynchrony indices (LVEF, EDV, ESV, SD, LL) as dependent variables. For each outcome, Type III sums of squares were used to evaluate main effects, and Least Significant Difference (LSD) post-hoc comparisons were applied to explore pairwise differences between diagnosis groups. Associations among continuous resting and stress parameters were examined with Pearson correlation coefficients and visualized using a correlation heatmap. To evaluate predictive factors, a multivariable logistic regression model including key LV metrics and demographic variables was fitted, and model performance was assessed using 10-fold cross-validation accuracy, calibration tests (Hosmer–Lemeshow and Pearson χ^2^), discrimination (ROC-AUC), pseudo-R^2^, and likelihood ratio testing.

Descriptive statistics, including mean, Standard Deviation (SD), and range, were calculated for the entire population and separately by sex (M and F) and age group (Young, Middle, Senior). Two approaches for normal range estimation were applied: Mean ± 2SD, which captures approximately 95% of the population assuming a normal distribution, and Mean ± SD, providing an additional measure of variability. Welch's t-tests were used to compare mean cardiac parameters between each group and the normal reference (Diagnosis 1), stratified by age group, to account for unequal sample sizes and variances across subgroups.

Logistic regression models assessed predictors of normal versus abnormal LVEF-S and CAD diagnosis. CAD cases were validated using confirmatory tests, including coronary catheterization. Model performance was evaluated using cross-validation, goodness-of-fit tests (*e.g.*, Pearson, Hosmer-Lemeshow), and confidence intervals to ensure reliability.

Missing data were minimal and addressed using multiple imputation (20 imputations) for variables with missing values, followed by pooling using Rubin's rules. Sensitivity analyses comparing imputed results to complete-case analyses revealed no meaningful differences in key quantitative parameters (LVEF-S, EDV-S, ESV-S, SV-S, Mean Angle-S, SD°-S, and their rest equivalents).

### 2.10 Quality Assurance

The dataset was cleaned to remove missing values and inconsistencies to ensure data integrity. Analyses were conducted using Minitab^®^ Statistical Software and Python libraries (pandas, seaborn, matplotlib). Two nuclear medicine consultants approved all diagnoses, while two nuclear medicine technologists verified image quality and processing accuracy.

### 2.11 Validation Strategy

Validation of gated SPECT MPI-derived functional parameters was conducted using two complementary, clinically accepted approaches. First, left ventricular ejection fraction, ventricular volumes, and dyssynchrony indices were compared against established clinical reference ranges from the literature to confirm physiological plausibility and expected sex-related trends. Second, internal clinical discrimination was evaluated by examining differences in these parameters across predefined diagnostic groups, with consistent and significant group separation considered as evidence of construct validity. Direct validation against invasive or multimodality reference standards was not possible due to ethical and practical considerations.

## 3 RESULTS

### 3.1 Sample Size Justification

A total of 932 cases were included in this study, which exceeds the minimum requirement determined by power estimation. A priori power analysis (α = 0.05, power = 0.80, medium effect size *f* = 0.25) indicated that a minimum of approximately 158 subjects would be sufficient to detect significant group differences using ANOVA. Therefore, the selected sample size provided ample statistical power and reduced the likelihood of Type II error. Furthermore, the cohort size aligns with or exceeds that used in comparable myocardial perfusion SPECT normative studies [14, 16], ensuring robustness and representativeness of the derived reference values.

### 3.2 Descriptive Statistics

Descriptive statistics were computed for 932 participants under Stress (S) and 462 under Rest (R) conditions, yielding 932 readings for each variable. Mean Left Ventricular Ejection Fraction (LVEF) remained stable at 68% for both conditions. Mean End-Diastolic Volume (EDV) was 95.10 ml (S) and 104.76 ml (R), while End-Systolic Volume (ESV) averaged 34.69 ml (S) and 40.10 ml (R). Stroke Volume (SV) measured 60 ml (S) and 64.6 ml (R). Dyssynchrony parameters showed a mean angle of 49.60° (S) and 50.40° (R). Standard deviation of angles (SD°) was 5.67° (S) and 5.45° (R), with slight differences in Upper Limit (UL) and Lower Limit (LL) values between stress and rest conditions. Percentile values (25th, 75th, and 95th) displayed similar patterns (Table **2** and Appendix **1**).

**Fig (3):**
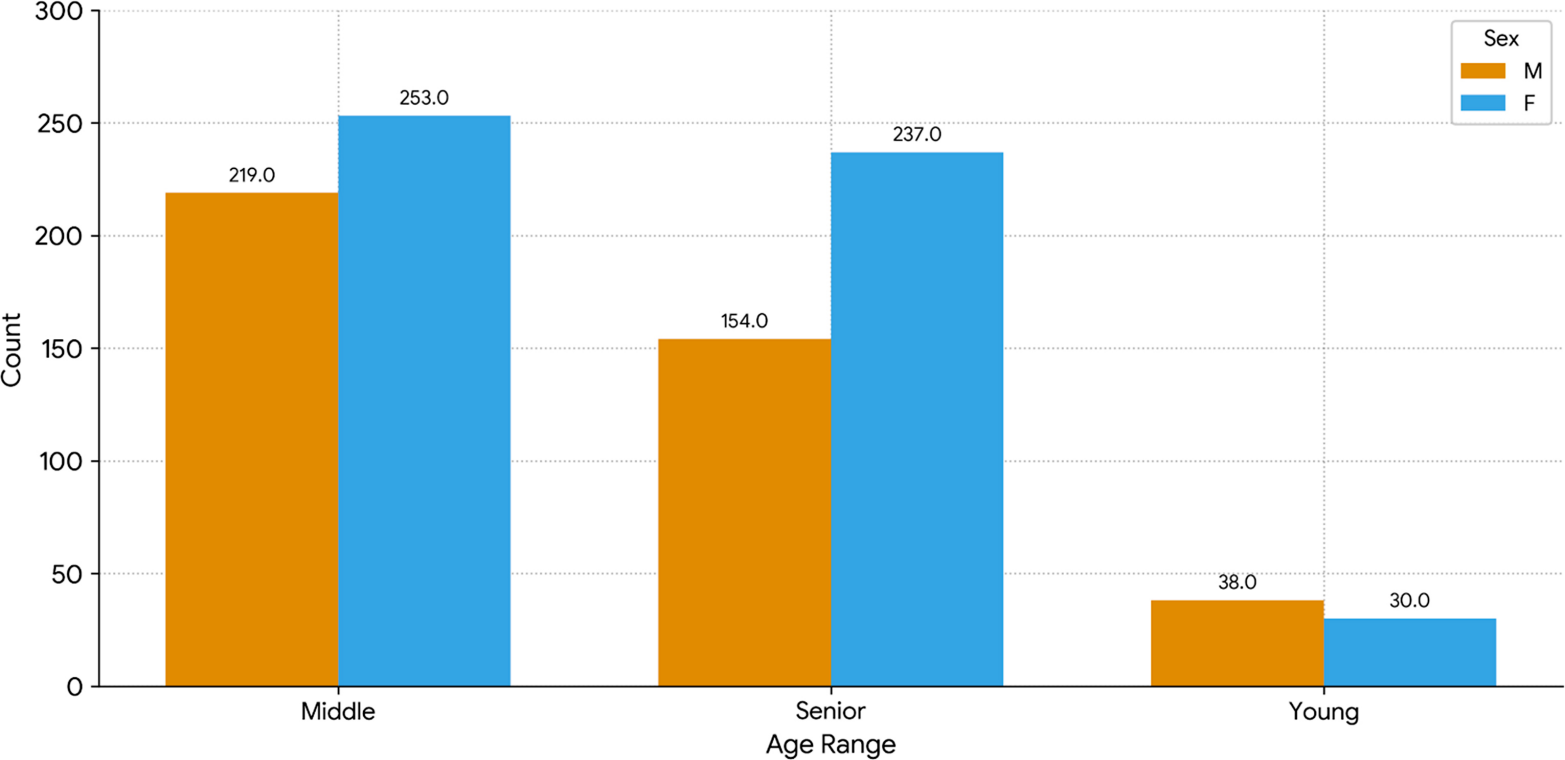
Age by variate (Sex) distribution. **M** is male and **F** is female.

The dataset included 520 females (55.77%) and 412 males (44.23%), with percentages based on a total of 932 participants. Participant demographics showed a mean height of 162.19 cm, a weight of 84.76 kg, and an age of 56.73 years. Height ranged from 93 to 189 cm, weight from 51 to 100 kg, and age from 17 to 88 years. Median values were 161 cm, 84 kg, and 57 years. Most participants fell within the Middle and Senior age categories (Fig. **3**). Of the cohort, 757 participants were normal, while 175 were abnormal. Diagnoses were verified through second opinions from nuclear medicine and radiology consultants.

### 3.3 Diagnosis Analysis

At stress, mean LVEF progressively declined across diagnostic groups (0.72 in normal, 0.66 in ischemic, and 0.51 in infarcted cases), accompanied by stepwise increases in EDV and ESV, indicating progressive ventricular dilation and systolic impairment. Similar patterns were observed at rest, with infarction cases showing the largest EDV (145 mL) and ESV (74 mL). Stroke Volume (SV) showed modest increases across groups, while dyssynchrony indices (SD°) rose markedly from normal to infarcted patients (5.4°, 5.8°, and 8.5° at stress), reflecting worsening mechanical discoordination with disease severity. Mean phase angles remained stable across groups, suggesting that the primary change involved phase dispersion rather than timing (Table **3**).

### 3.4 Comparative Analysis: Male *vs.* Female

Significant differences were observed between males and females across various parameters, diagnoses, and age groups. For the Middle age group in Diagnosis 1 (normal population), LVEF-S exhibited significant differences (T-Statistic = -9.27, *p* < 0.0001), with EDV-S, ESV-S, and SV-S also demonstrating significantly higher values in males (Tables **4**-**6**). Mean Angle-S and SD◦-S were also significantly different (*p* = 0.0072 and *p* = 0.0224, respectively). For Diagnosis 2, middle-aged participants displayed significant differences in LVEF-S (*p* = 0.027), EDV-S (*p* = 0.035), and ESV-S (*p* = 0.018). Diagnosis 5 showed significant differences in EDV-S, ESV-S, and SD◦-S (*p* = 0.0452, 0.0463, and 0.0029, respectively). In Diagnosis 3, EDV-S, ESV-S, and SV-S displayed significant differences (T-Statistics = 3.82, 2.52, and 5.25; *p* = 0.0002, 0.0133, and <0.0001, respectively).

For the Young age group in Diagnosis 4, EDV-S, ESV-S, SV-S, Mean Angle-S, and SD◦-S showed significant differences (*p*-values ranged from 0.0007 to 0.0365). The independent t-tests comparing diseased groups (Diagnoses 2, 3, 4, and 5) with the normal group (Diagnosis 1) revealed significant differences across cardiac parameters, with the details provided in Table **4** and Appendix **1**.

### 3.5 Age-Stratified Analysis

Cardiac function parameters were analyzed by age and sex within the Kuwaiti population. Overall mean LVEF-S was 68%, with a normal range of 55–82% (mean ± SD). Mean EDV-S was 95 mL, ranging from 53–137 mL; ESV-S averaged 35 mL (2–67 mL), and SV-S averaged 60 mL (44–77 mL). Females demonstrated higher LVEF-S values (72%) compared to males (63%). EDV-S was lower in females (80 mL) than in males (115 mL). ESV-S was also lower in females (25 mL) compared to males (47 mL), and SV-S showed a similar pattern, with females at 55 mL and males at 68 mL.

Age-specific results showed that young females had the highest LVEF-S (73%; range 62–83%), followed by middle-aged (72%; range 59–84%) and senior females (72%; range 60–84%). Among males, LVEF-S was highest in middle-aged individuals (65%), while seniors and young males both averaged 62%. For dyssynchrony parameters, mean angle at stress (Mean Angle-S) was 50° (range: 41–59°), with SD°-S at 5° (range: 2–8°). Females showed a lower Mean Angle-S (49°) and higher SD°-S (6°) compared to males (51° and 5°, respectively). Detailed results are presented in Table **5** and Appendix **1**.

### 3.6 Between-Subjects Effects

The between-subjects analysis proved that diagnosis affected all resting SPECT MPI parameters significantly and consistently (LVEF-R, EDV-R, ESV-R, SD-R, and LL-R; all *p* < 0.001). This implies that patients grouped into various diagnostic categories had significant disparities in both ventricular volumes and dyssynchrony indices at rest. Conversely, age range was found to have no significant impact on any of the measured parameters (*p* >0.001), indicating that even the resting volumetric and dyssynchrony indices do not change significantly between age groups in this cohort. Sex was a significant factor in volumetric indexes LVEF-R, EDV-R, and ESV-R (all *p* <0.001), with males and females having different values in these variables, but sex did not have any significant impact on the parameters of dyssynchrony SD-R or LL-R (Table **6**).

LSD post-hoc analysis showed significant pairwise differences in most resting volumetric and dyssynchrony parameters across diagnostic groups. LVEF-R was significantly higher in Diagnosis 1 than Diagnosis 2 (mean difference = 3.11%, 95% CI 0.51–5.72) and Diagnosis 3 (16.33%, 95% CI 11.93–20.73), with Diagnosis 3 showing the lowest values. EDV-R demonstrated a graded increase, being significantly higher in Diagnosis 3 than Diagnosis 1 (49.72 mL, 95% CI 36.14–63.30) and Diagnosis 2 (41.60 mL, 95% CI 28.57–54.62), while Diagnosis 2 also exceeded Diagnosis 1 (8.12 mL, 95% CI 0.10–16.14). ESV-R was significantly greater in Diagnosis 3 compared with Diagnosis 1 (40.95 mL, 95% CI 30.25–51.66) and Diagnosis 2 (35.73 mL, 95% CI 25.47–45.99), with no significant difference between Diagnosis 1 and Diagnosis 2. Both SD-R and LL-R were significantly higher in Diagnosis 3 than in Diagnosis 1 and Diagnosis 2 (all *p* < 0.001), whereas no significant dyssynchrony differences were observed between Diagnosis 1 and Diagnosis 2.

The between-subjects analysis of the stress SPECT MPI parameter revealed that diagnosis exerted a significant and statistically significant influence on all stress-based measures of volumes and dyssynchrony, such as LVEF-S, EDV-S, ESV-S, SD-S, and LL-S (all *p* <0.001). This implies that the membership of diagnostic groups is a significant predictor of left ventricular functioning and mechanical synchrony in response to stress. Conversely, there was no significant age effect on most stress parameters (*p* >0.001), but a small, statistically significant effect on EDV-S (p =0.027), indicating a small amount of age-related variation in stress volumes. Sex showed a significant impact on all the large stress volumetric indices, LVEF-S, EDV-S, and ESV-S (all *p* < 0.001), and significantly influenced the dyssynchrony parameter SD-S (*p* =0.006) and the parameter of the dyssynchrony LL-S (*p* =0.024). These results suggest that there is a significant sex difference in cardiac response to stress (Table **7**).

LSD post-hoc analysis demonstrated significant pairwise differences in all stress SPECT MPI volumetric and dyssynchrony parameters across diagnostic groups (Table **4**). LVEF-S was significantly higher in Diagnosis 1 than Diagnosis 2 (mean difference = 5.99%, 95% CI 4.35–7.64) and Diagnosis 3 (20.38%, 95% CI 16.91–23.85), with Diagnosis 3 exhibiting the lowest stress ejection fraction. Stress ventricular volumes showed a graded increase, with EDV-S significantly higher in Diagnosis 3 compared with Diagnosis 1 (57.75 mL, 95% CI 47.20–68.31) and Diagnosis 2 (40.88 mL, 95% CI 30.04–51.71), while Diagnosis 2 also exceeded Diagnosis 1 (16.88 mL, 95% CI 11.87–21.88). Similar patterns were observed for ESV-S, with Diagnosis 3 demonstrating markedly higher values than Diagnosis 1 (50.42 mL, 95% CI 42.14–58.70) and Diagnosis 2 (38.28 mL, 95% CI 29.77–46.78). Stress-induced dyssynchrony was significantly greater in Diagnosis 3 than in Diagnosis 1 and Diagnosis 2 for both SD-S and LL-S (all *p* < 0.001), with smaller but significant differences also observed between Diagnosis 2 and Diagnosis 1.

### 3.7 Resting Dyssynchrony and Stress LVEF Correlations by Sex and Diagnosis

Resting phase dyssynchrony indices showed significant and directionally consistent correlations with stress left ventricular ejection fraction (LVEF-S) across diagnostic and sex groups.

In normal males and females, LVEF-S was strongly and inversely correlated with SD◦-R (r = −0.714, 95% CI [−0.813, −0.574], *p* < 0.001), UL-R (r = −0.569, 95% CI [−0.710, −0.385], *p* < 0.001), and upper phase percentiles (*e.g.*, at 95-R, r = −0.653, 95% CI [−0.770, −0.492], *p* < 0.001), while LL-R was positively correlated (r = 0.617, 95% CI [0.445, 0.745], *p* < 0.001).

Among ischemic patients, males exhibited moderate to strong negative correlations for SD◦-R (r = −0.645, 95% CI [−0.732, −0.537], *p* < 0.001) and at 95-R (r = −0.628, 95% CI [−0.718, −0.516], *p* < 0.001), with LL-R showing a positive association (r = 0.597, 95% CI [0.479, 0.693], *p* < 0.001). Female ischemic patients displayed weaker but still significant negative correlations for UL-R (r = −0.390, 95% CI [−0.535, −0.223], *p* < 0.001) and at 95-R (r = −0.273, 95% CI [−0.434, −0.095], *p* = 0.003).

In infarction patients, the relationships intensified in males, SD◦-R (r = −0.583, 95% CI [−0.788, −0.261], *p* = 0.001) and UL-R (r = −0.580, 95% CI [−0.787, −0.257], *p* = 0.002) showed strong inverse associations, while LL-R correlated positively (r = 0.627, 95% CI [0.324, 0.813], *p* < 0.001). In contrast, female infarction patients showed only one strong positive correlation for LL-R (r = 0.608, 95% CI [0.180, 0.842], *p* = 0.010).

Overall, these findings demonstrate that increasing resting dyssynchrony, particularly elevated SD◦-R and UL-R, is consistently associated with reduced LVEF-S, with the magnitude of these associations increasing from normal to ischemic to infarcted states and being more pronounced in males than in females.

### 3.8 Logistic Regression and Predictive Modelling

The logistic regression model identified several statistically significant predictors. LVEF-S showed a positive and significant association with the outcome (β = 0.449, SE = 0.117, z = 3.85, *p* < 0.001), with the 95% CI ranging from 0.221 to 0.678. Among the volumetric parameters, EDV-R demonstrated a significant negative coefficient (β = −4898.35, SE = 416.47, z = −11.76, *p* < 0.001), whereas ESV-R (β = 3768.58, SE = 320.37, z = 11.76, *p* < 0.001) and SV-R (β = 1935.97, SE = 164.62, z = 11.76, *p* < 0.001) showed significant positive effects; all corresponding confidence intervals excluded zero. Other dyssynchrony-related predictors, including SD-R, UL-R, LL-R, at25-R, at75-R, and at95-R, did not reach statistical significance (*p* > .05). Regarding demographic variables, the Senior age group showed a significant negative association (β = −0.411, SE = 0.166, z = −2.48, *p* = 0.013), while the Young age group was not significant (*p* = .530). Sex was also a significant predictor, with male participants showing a negative association (β = −0.423, SE = 0.170, z = −2.49, *p* = 0.013). The model intercept was statistically significant (β = 1.806, SE = 0.173, z = 10.45, *p* < 0.001).

**Fig (4):**
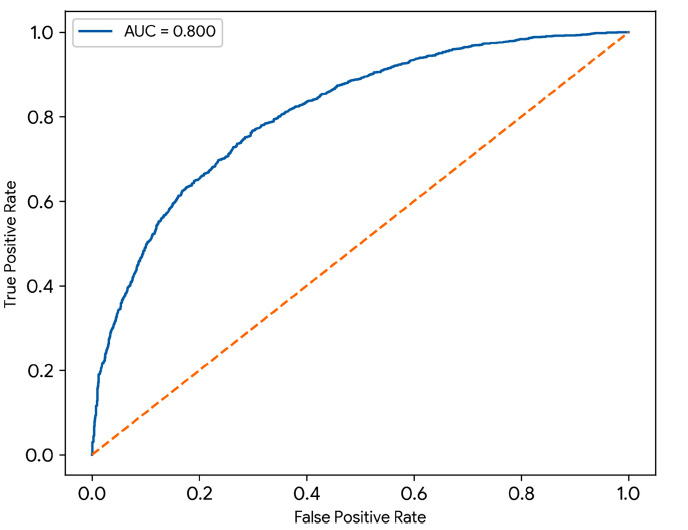
Receiver operating characteristic (ROC) curve demonstrating the discriminative performance of the logistic regression model incorporating resting and stress SPECT-derived volumetric and dyssynchrony parameters and demographic variables.

The cross-validation that was conducted 10 times produced a mean accuracy of 0.65 (SD = 0.04), which shows predictive consistency across folds. The model could be calibrated, as indicated by Hosmer Lemeshow 2(5) = 5.59 and a *p*-value = 0.69, which is not significant, and thus no indication of bad fit. Equally, Pearson χ^2^ test (938.67, *p* = 0.31) was also significant to indicate sufficient model fit. The ability to discriminate, evaluated using the receiver operating characteristic curve, yielded an AUC of 0.80, indicating good separation between diagnostic groups (Fig. **4**). The model described about 21.4% of the variance based on the McFadden pseudo R^2^. The last model had a log-likelihood of -496.43 against the null log-likelihood of -631.86, and the likelihood ratio test showed that the model was highly significant more than the null(*p* < 0.001).

## 4 DISCUSSION

This study established sex- and age-specific normal reference ranges for left ventricular volumetric and dyssynchrony parameters derived from gated SPECT Myocardial Perfusion Imaging (MPI) in a large Kuwaiti population. A total of 932 participants were analyzed under stress conditions, and 462 under rest conditions. Mean LVEF was 68% in both states, with corresponding mean EDV of 95.10 mL (stress) and 104.76 mL (rest), ESV of 34.69 mL (stress) and 40.10 mL (rest), and SV of 60 mL (stress) and 64.6 mL (rest). Dyssynchrony parameters demonstrated mean phase angles of 49.6° (stress) and 50.4° (rest), with SD◦ values of 5.67° (stress) and 5.45° (rest). The cohort comprised 520 females (55.77%) and 412 males (44.23%), with a mean age of 56.7 years; most participants were middle-aged (n = 472) or senior (n = 391). Of the total cohort, 572 were classified as normal and 385 as abnormal (ischemia or infarction), with diagnoses independently confirmed by nuclear medicine and radiology consultants.

Significant sex-related differences in left ventricular function were observed, consistent with established literature [18]. Males exhibited higher volumetric parameters (EDV-S, ESV-S, SV-S), whereas females demonstrated higher LVEF-S and lower EDV-S and ESV-S, particularly in younger and middle-aged groups. Similar volumetric sex differences have been reported previously [18-20]. These differences are not merely statistical but clinically critical for defining accurate, sex-adjusted normal limits, improving diagnostic precision, and guiding individualized interpretation of cardiac imaging results [18, 21]. Dyssynchrony parameters (Mean Angle-S and SD◦-S) also differed significantly by sex, underscoring physiological distinctions in ventricular mechanics and reinforcing the need for sex- and age-specific reference ranges in quantitative MPI interpretation [1].

Age-stratified analyses showed that females consistently maintained higher LVEF-S values (mean ~72%) compared with males (mean ~63%). Female LVEF-S remained stable across age groups (72–73%), whereas male LVEF-S peaked in middle age (~65%) before declining slightly in older groups. Males consistently exhibited larger EDV and ESV volumes across all ages. These findings align with known physiological sex differences and age-related myocardial remodeling patterns reported in population-based cardiac imaging studies [1-3, 21]. For the Kuwaiti population, these age- and sex-specific patterns are particularly important, as failure to account for them may lead to misclassification of normal studies and biased diagnostic thresholds [22]. Large contemporary CMR reference datasets and meta-analyses similarly recommend sex-adjusted ranges for LV volumes and EF, consistent with our observations [22]. Applying sex- and age-specific normal limits, therefore, enhances quantitative MPI interpretation and clinical decision-making [7, 16].

Clinical diagnosis exerted a strong and consistent influence on both resting and stress-induced LV volumetric and dyssynchrony parameters (LVEF, EDV, ESV, SD, LL; all *p* < 0.001). Post-hoc analyses demonstrated a graded pattern of cardiac impairment across diagnostic groups. Diagnosis 3 (infarction) exhibited the lowest LVEF and the highest EDV and ESV at rest and stress, reflecting severe systolic dysfunction and advanced ventricular dilatation, alongside markedly elevated dyssynchrony indices indicating impaired mechanical coordination. Diagnosis 1 demonstrated preserved systolic function and minimal dyssynchrony, while Diagnosis 2 (ischemia) represented an intermediate phenotype with moderate volumetric enlargement and mild dyssynchrony. These findings support the value of SPECT MPI in distinguishing between diagnostic subgroups using combined volumetric and mechanical indices and align with prior studies showing progressive ventricular remodeling and dyssynchrony in complex cardiac pathology.

Mechanical discoordination observed in Diagnosis 3, even at rest, suggests that dyssynchrony may occur before overt volumetric changes in certain disease states. The greater differences observed under stress further emphasize the added diagnostic value of stress imaging in detecting latent functional impairment not apparent at rest.

An apparent paradox was noted in some subgroups, where stress LVEF-S appeared elevated despite disease, alongside smaller EDV-S and ESV-S. This phenomenon can be explained by two mechanisms. First, the small-heart effect inherent to gated SPECT may result in overestimation of LVEF and underestimation of LV volumes, particularly in individuals with small ventricles [23-25]. Second, physiological stress can enhance contractility and reduce LV cavity size in patients without extensive ischemia, leading to higher post-stress LVEF and lower ESV [26]. Sex- and size-related factors further influence this relationship, as women typically have smaller LV volumes and higher EF values [7, 27]. Therefore, this apparent paradox reflects combined physiological and methodological influences rather than a true contradiction in cardiac performance.

Resting dyssynchrony indices showed consistent and directionally meaningful correlations with stress LVEF-S across diagnostic and sex categories. In both men and women, higher resting SD°-R and UL-R were associated with lower stress LVEF-S, while LL-R showed a positive association with systolic performance. These relationships were more evident in ischemic and infarcted groups, particularly among males, suggesting that increased resting mechanical dyssynchrony corresponds to reduced systolic reserve under stress. These findings align with evidence that phase SD° and bandwidth derived from gated SPECT reflect ventricular synchrony and contractile efficiency and are linked to adverse outcomes [28].

Binary logistic regression identified volumetric parameters and demographic factors, rather than resting dyssynchrony indices, as the main independent predictors of abnormal stress left ventricular function. Higher stress LVEF-S was positively associated with the modeled outcome, confirming the central role of stress systolic performance. Among resting volumetric measures, EDV-R showed a significant inverse association, while ESV-R and SV-R showed strong positive associations, indicating that baseline ventricular size and stroke volume contribute meaningfully to stress functional status. In contrast, resting dyssynchrony parameters, including SD-R, UL-R, LL-R, and phase percentiles, did not retain independent significance after multivariable adjustment, suggesting that their predictive contribution may be secondary to volumetric determinants. Demographic effects remained relevant, with male sex and senior age independently associated with abnormal stress function. The model showed good discrimination (AUC = 0.80), acceptable calibration (Hosmer–Lemeshow p = 0.69), and stable cross-validation performance, explaining approximately 21% of the variance. These findings indicate that conventional volumetric indices remain the dominant predictors of stress systolic impairment in this cohort, while dyssynchrony measures may provide complementary, but not independent, prognostic information within multivariable models.

Clinically, these findings highlight that baseline mechanical dyssynchrony contributes meaningfully to impaired systolic response under stress [5, 29], independent of diagnostic category and sex. This supports incorporating phase-derived synchrony indices into routine quantitative SPECT MPI to improve risk stratification and diagnostic accuracy. Over the past three decades, SPECT MPI has been established as a cornerstone modality for evaluating coronary artery disease and myocardial dysfunction, with endorsement from major professional societies (ASNC, EANM, SNMMI, BNMS, CANM, ARCCNM, JSNM, IAEA) [30-32]. However, variability in functional measurements due to differences in segmentation algorithms, reconstruction techniques, and acquisition protocols remains a challenge [33-35].

Rather than revalidating technical acquisition and reconstruction factors, the present study focuses on establishing standardized, population-specific reference ranges for conventional volumetric and phase-based synchrony measures derived from gated SPECT MPI. The findings show that reliance on LVEF alone may miss clinically relevant functional information. Combining LVEF with ventricular volumes and dyssynchrony indices provides a more comprehensive assessment of left ventricular performance and improves diagnostic interpretation, prognostic evaluation, and clinical risk stratification [36-41].

Future work should focus on external validation of the proposed sex- and age-specific reference ranges in independent, multi-center cohorts to evaluate their robustness and transferability across different clinical settings. Generalizability studies are needed to assess performance across populations with varying demographic profiles and disease prevalence. Direct comparison with clinical gold standards, including expert consensus interpretation and complementary functional modalities such as echocardiography or invasive hemodynamic measurements where available, will be essential to confirm clinical validity. Additionally, harmonization studies across imaging systems, reconstruction protocols, and analysis software are needed to quantify system-dependent variability and refine standardized quantitative thresholds. Together, these steps will support broader clinical adoption and strengthen the role of quantitative SPECT MPI in routine patient assessment.

## 5 LIMITATIONS

This retrospective, single-center study may be subject to selection bias, although participant demographics closely matched international stress-testing populations. The findings primarily reflect the Kuwaiti population, where factors such as obesity, diabetes, and hypertension may influence cardiac performance and ventricular remodeling, limiting generalizability. Analyses were performed using a single software platform (Myovation^®^), and differences in segmentation algorithms across other tools (*e.g.*, Emory Cardiac Toolbox^®^, QPS^®^, Corridor4DM^®^) may affect parameter comparability. The “normal” cohort definition, based on clinical reports and absence of cardiovascular history, may introduce incorporation bias since these same interpretations informed parameter validation. Accordingly, the reported ranges should be regarded as clinically derived baselines rather than absolute biological standards. Direct validation against invasive reference measures was not feasible due to ethical and practical considerations. Future multicenter studies and validation against independent expert or multimodality assessments are recommended to confirm these findings.

## CONCLUSION

This study presents clinically derived reference ranges for myocardial perfusion and functional parameters across age, sex, and diagnostic groups, offering preliminary benchmarks for individualized cardiac assessment. The integration of volume- and phase-based indices with demographic and physiological variables (such as age and weight) demonstrates their potential value for interpreting resting data and predicting stress-induced LVEF changes.

Through consistent statistical modeling and internal cross-validation, the findings contribute to strengthening population-specific normative data. However, further multicenter research is needed to verify generalizability and to evaluate how acquisition or reconstruction variability may influence parameter stability. The framework outlined in this study may also inform subsequent radiomics and machine-learning studies aimed at refining diagnostic thresholds and improving cardiac function characterization..

## Figures and Tables

**Fig (2):**
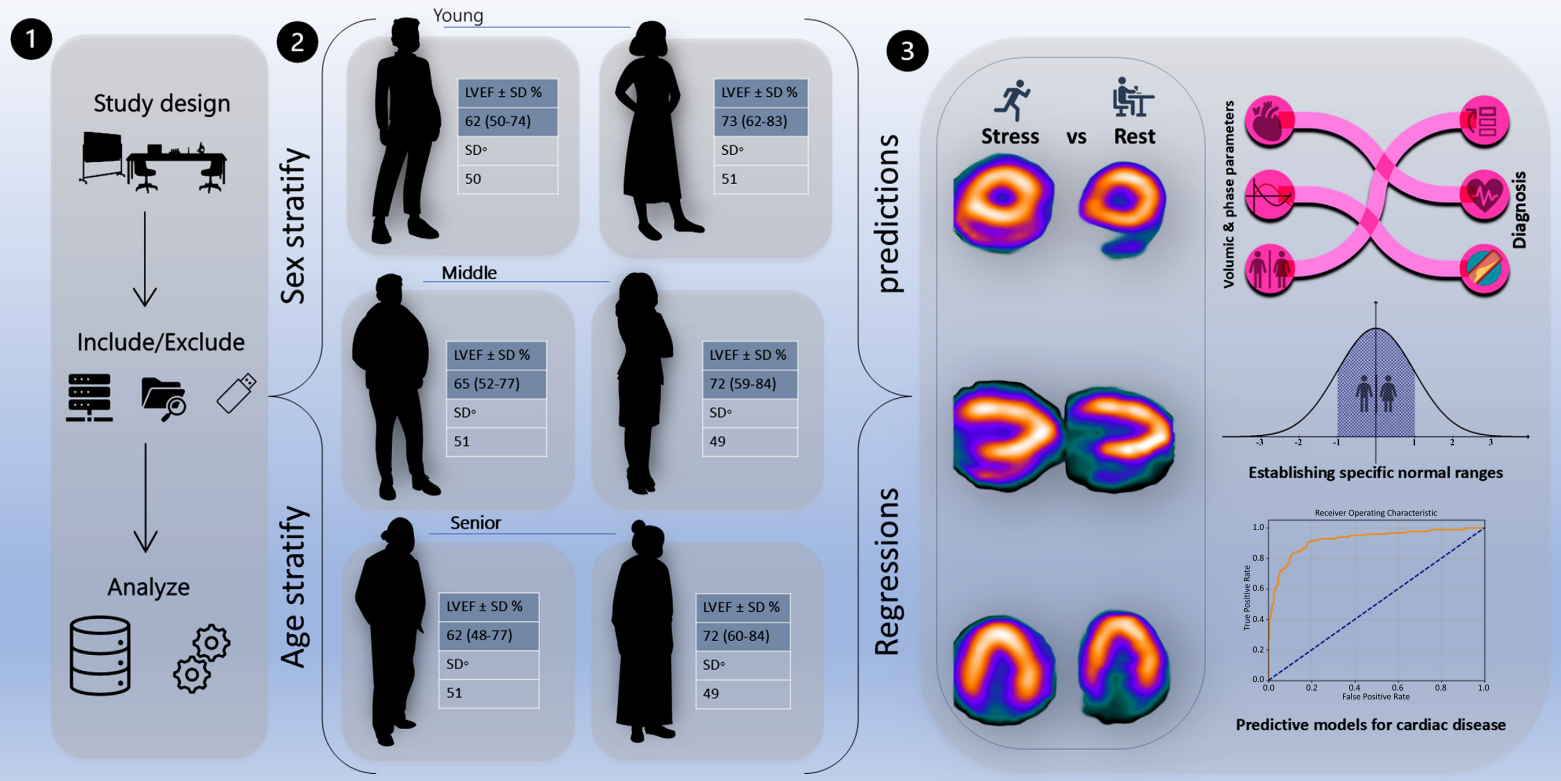
A graphical abstract representing the methodology of this study. **1** represent the study initial phase, **2** represent the classification strategy and **3** represent the data analysis phase.

**Table 1:** The recoded values of diagnosis and history during statistical analysis. Only 1 is considered normal.

Recode	Normal	Diagnosis Mapping Code
**1**	Yes	No significant ischemia, no significant scintigraphic evidence, no definite evidence of ischemia, no stress-induced ischemia, Normal study, No definite scintigraphic evidence, No scintigraphic evidence, adequate perfusion in left ventricular myocardium, no evidence of myocardial ischemia, No definite scintigraphic evidence to suggest significant stress-induced ischemia on present study.
**2**	No	Stress-induced myocardial ischemia, evidence of ischemia, ischemia in the lateral wall, ischemia in the anteroseptal wall, ischemia in the anterior wall, reversible perfusion defect, wall ischemia.
**3**	No	Myocardial infarction, peri-infarct ischemia, fixed perfusion defect, evidence of myocardial infarction, atherosclerotic, coronary calcifications are present, perfusion defect, apical wall ischemia, large, fixed perfusion defect, fixed perfusion defect, abnormal study, perfusion defect, suggestive of stress-induced ischemia.

**Table 2:** Overall descriptive statistics for all SPECT MPI parameters. LVEF, and EDV/ESV/SV are presented as mean fractions and mean volumes (ml), respectively. Phase analysis dyssynchrony parameters: Mean Angle indicates the average onset of contraction; SD° reflects global dyssynchrony; UL and LL are the upper and lower phase limits; 25°, 75°, and 95° represent phase percentiles describing the distribution of ventricular contraction timing.

		Volumetric				Dyssynchrony Parameters						
Statistic		**LVEF^1^**	**EDV^2^**	**ESV^3^**	**SV^4^**	**Mean Angle**	**SD^◦(5)^**	**UL^6^**	**LL^7^**	**at 25^◦(8)^**	**at 75^◦(9)^**	**at 95^◦(10)^**
Mean	S	0.68	95	35	60	49.6	5.7	66	34.3	3.7	11.8	22.5
	R	0.67	104	40	65	50.4	5.4	66.3	34.8	3.7	11.5	21.4
Std	S	0.13	42.	33	17	8.5	3	12.8	12.9	2.5	6.6	13.4
	R	0.14	47	36	18	8.4	3.2	13.4	13.17	2.5	6.7	13.8
Min	S	0.19	15	0	-60	5.3	1	0	0	1	0	4
	R	0.21	25	1	11	31.4	1	39	0	1	2	4
25%	S	0.62	68	16	49	41.9	3.6	57	27	2	8	14
	R	0.21	25	1	11	31.4	1	39	0	1	2	4
50%	S	0.71	86	25	59	50.5	5	66	35	2	10	20
	R	0.59	73	17	52	43.3	3.4	58	27	2	8	14
75%	S	0.77	111	40	70	56.8	6.9	72	44	4	14	26
	R	0.59	73	17	52	43.3	3.4	58	27	2	8	14

**Notes:** ^1^Left Ventricular Ejection Fraction, ^2^End-Diastolic Volume, ^3^End-Systolic Volume, ^4^Stroke Volume, ^5^Standard Deviation of Phase, ^6^Upper Limit, ^7^Lower Limit, and Phase Angles at the ^(8)^25th, ^(9)^75th, and ^(10)^95th percentiles represent key quantitative parameters used to evaluate left ventricular function and mechanical synchrony in myocardial perfusion imaging.

**Table 3:** Descriptive statistics by diagnosis. LVEF and EDV/ESV/SV are shown as mean fractions and volumes (in milliliters (mL)). SD° denotes phase angle variability. N = observations; N* = missing; SE Mean = standard error; StDev = standard deviation; Q1–Q3 = distribution percentiles. Suffix -S represents stress, and -R represents rest.

**Variable**	Diagnosis	N	N*	Mean	SE Mean	StDev	Min	Q1	Median	Q3
**LVEF^1^-S**	1	547	0	0.72	0.0	0.11	0.19	0.67	0.73	0.79
	2	334	0	0.66	0.01	0.14	0.20	0.58	0.68	0.74
	3	51	0	0.51	0.02	0.17	0.24	0.38	0.49	0.65
**EDV^2^-S (mL)**	1	547	0	86	1.5	35	15	64	80	100
	2	334	0	103	2.4	43	39	74	93	119
	3	51	0	144	8.6	62	34	100	129	176
**ESV^3^-S (mL)**	1	547	0	28	1.1	26	0	14	22	32
	2	334	0	40	1.8	33	4	19	29	49
	3	51	0	78	7.4	53	2	33	74	101
**SV^4^-S (mL)**	1	547	0	58	0.7	16	–60	48	57	67
	2	334	0	63	1.0	17	20	51	61	74
	3	51	0	66	2.4	17	28	53	66	79
**Mean Angle-S(°)**	1	547	0	49.5	0.4	8.9	5.3	41.8	50.4	57.0
	2	334	0	49.6	0.4	8.0	34	42	50	56
	3	51	0	50.2	1.2	8.7	33	42	51	58
**SD°^(5)^-S**	1	547	0	5.4	0.1	2.7	1.0	3.6	4.8	6.7
	2	334	0	5.8	0.2	3.1	1.7	3.6	5.1	7.0
	3	51	0	8.5	0.6	4.2	2.5	5.0	7.8	11.5
**LVEF-R**	1	161	386	0.70	0.01	0.13	0.27	0.65	0.72	0.78
	2	257	77	0.67	0.01	0.14	0.21	0.60	0.70	0.78
	3	44	7	0.54	0.02	0.16	0.22	0.41	0.53	0.68
**EDV-R (mL)**	1	161	386	96	3.4	43	25	68	88	112
	2	257	77	104	2.6	42	26	74	92	128
	3	44	7	145	9.3	62	34	105	144	174
**ESV-R (mL)**	1	161	386	33	2.6	33	1	15	24	36
	2	257	77	39	2.0	32	3	17	28	47
	3	44	7	74	7.3	48	5	34	69	107
**SV-R (mL)**	1	161	386	62	1.3	17	17	51	61	73
	2	257	77	65	1.1	18	11	53	63	76
	3	44	7	71	3.6	24	29	55	66	82
**Mean Angle-R(°)**	1	161	386	50.7	0.7	8.9	32	43	53	58
	2	257	77	50.1	0.5	8.2	31	43	52	57
	3	44	7	51.1	1.2	7.6	35	45	53	57
**SD°-R**	1	161	386	5.0	0.2	2.7	1.3	3.4	4.2	5.8
	2	257	77	5.3	0.2	3.0	1.0	3.3	4.8	6.3
	3	44	7	7.9	0.7	4.8	2.2	4.2	6.3	10.1

**Notes:** ^1^Left Ventricular Ejection Fraction, ^2^End-Diastolic Volume, ^3^End-Systolic Volume, ^4^Stroke Volume and ^5^Standard Deviation of Phase.

**Table 4:** A Comparison of stress-phase and volumetric parameters between diagnostic groups using Welch’s t-test (Diagnosis 1 = reference group). The table summarizes significant mean differences in left-ventricular function and dyssynchrony indices (LVEF-S, EDV-S, ESV-S, SD◦-S, and LL-S) across age-stratified subgroups, indicating that both ischemic (Diagnosis 2) and infarcted (Diagnosis 3) cohorts exhibit greater volumetric enlargement and reduced systolic performance relative to normal subjects. Suffix -S represents stress, and -R represents rest.

Variable	Diagnosis	Age Range	T-statistic	*p*-value
**EDV^1^-S**	2	Young	4.16	0.0002
**EDV-S**	2	Middle	5.43	<0.0001
**EDV-S**	2	Senior	2.37	0.0187
**EDV-S**	3	Middle	5.9	<0.0001
**EDV-S**	3	Senior	3.9	0.0005
**ESV^2^-S**	2	Young	3.4	0.0019
**ESV-S**	2	Middle	4.61	<0.0001
**ESV-S**	2	Senior	3.13	0.0020
**ESV-S**	3	Middle	5.77	<0.0001
**ESV-S**	3	Senior	4.07	0.0003
**LL^3^-S**	3	Middle	-3.43	0.0027
**LL-S**	3	Senior	-3.23	0.0029
**LVEF^4^-S**	2	Young	-3.13	0.0037
**LVEF-S**	2	Middle	-4.88	<0.0001
**LVEF-S**	2	Senior	-4.63	<0.0001
**LVEF-S**	3	Middle	-9.91	<0.0001
**LVEF-S**	3	Senior	-4.74	<0.0001
**SD◦^(5)^-S**	2	Senior	2.35	0.0196
**SD◦-S**	3	Middle	3.76	0.0013
**SD◦-S**	3	Senior	3.47	0.0016
**SV^6^-S**	2	Young	4.02	0.0003
**SV-S**	2	Middle	4.77	<0.0001
**SV-S**	3	Middle	2.89	0.0088

**Notes:** ^1^End-Diastolic Volume, ^2^End-Systolic Volume, ^3^Lower Limit, ^4^Left Ventricular Ejection Fraction,, ^5^Standard Deviation of Phase and, ^6^Stroke Volume

**Table 5:** Show age-stratified left ventricle volume-based parameters. Normal ranges are expressed as (MEAN ± standard deviation (SD)) and (MEAN ± 2 standard deviation (2SD)). Y represents young, Mid is middle and S is senior. F is female, and M is male.

**CAT**		**LVEF^1^ MEAN ± SD (%)**	**LVEF^1^ MEAN ± 2SD (%)**	**EDV^2^ MEAN ± SD (%)**	**EDV^2^ MEAN ± 2SD (%)**	**ESV^3^ MEAN ± SD (%)**	**ESV^3^ MEAN ± 2SD (%)**	**SV^4^ MEAN ± SD (%)**	**SV^4^ MEAN ± 2SD (%)**
**ALL**	S	68 (55-82)	41-95	95 (53-137)	10-180	35 (2-67)	0-100	60 (44-77)	27-94
	R	67 (52-81)	38-96	105 (58-151)	11-198	40 (4-76)	0-112	65 (46-83)	28-101
**ALL (F)**	S	72 (60-84)	48-96	80 (50-109)	20-139	25 (4-46)	0-67	55 (39-70)	24-86
	R	72 (59-85)	46-98	85 (51-118)	16-153	27 (3-51)	0-75	57 (42-73)	26-89
**ALL (M)**	S	63 (50-77)	37-91	115 (67-162)	20-209	47 (8-87)	0-126	68 (52-84)	36-99
	R	62 (48-76)	33-91	124 (75-172)	26-221	52 (12-93)	0-133	71 (53-90)	35-108
**MID (F)**	S	72 (59-84)	47-96	83 (51-114)	20-145	26 (3-49)	0-72	57 (42-71)	28-85
	R	69 (55-83)	40-97	93 (56-130)	19-167	33 (3-62)	0-91	60 (45-75)	30-91
**S (F)**	S	72 (60-84)	48-97	76 (48-104)	20-133	23 (4-42)	0-61	53 (36-69)	20-86
	R	74 (63-86)	51-97	78 (48-108)	18-138	22 (4-41)	0-59	56 (40-71)	25-87
**Y (F)**	S	73 (62-83)	52-93	79 (51-107)	23-135	23 (7-40)	0-57	56 (40-71)	25-86
	R	74 (64-83)	55-92	76 (46-105)	17-135	22 (7-37)	0-52	54 (38-70)	22-86
**MID (M)**	S	65 (52-77)	38-91	113 (69-157)	25-201	45 (8-82)	0-118	68 (53-84)	37-99
	R	63 (49-77)	35-91	121 (75-168)	29-214	50 (11-89)	0-127	71 (54-89)	36-107
**S (M)**	S	62 (48-77)	33-91	115 (63-167)	10-219	50 (6-94)	0-139	65 (50-80)	35-95
	R	60 (45-74)	31-89	126 (72-180)	18-234	56 (13-100)	0-143	70 (50-89)	30-109
**Y (M)**	S	62 (50-74)	38-87	125 (81-168)	38-211	51 (20-82)	0-113	74 (54-94)	34-114
	R	65 (51-79)	37-93	129 (94-164)	59-199	50 (15-84)	0-118	79 (64-94)	50-108

**Notes:** ^1^Left Ventricular Ejection Fraction, ^2^End-Diastolic Volume, ^3^End-Systolic Volume, ^4^Stroke Volume

**Table 6:** Effects of clinical diagnosis, age range, and sex on resting left ventricular volumetric and dyssynchrony metrics: Tests of between-subjects effects.

**Source**	**Dependent Variable**	**Type III Sum of Squares**	**DF^1^**	**Mean Square**	**F**	***p*-value**
**Intercept**	LVEF-R	723301.6	1	723301.686	4167.935	<0.001
	EDV-R	2287516.7	1	2287516.729	1386.592	<0.001
	ESV-R	397810.5	1	397810.542	388.026	<0.001
	SD-R	6275.6	1	6275.621	648.673	<0.001
	LL-R	198817.4	1	198817.498	1195.987	<0.001
**Diagnosis**	LVEF-R	7547.5	2	3773.796	21.746	<0.001
	EDV-R	68824.9	2	34412.489	20.859	<0.001
	ESV-R	49695.	2	24847.618	24.236	<0.001
	SD-R	300.9	2	150.482	15.554	<0.001
	LL-R	3271.8	2	1635.910	9.841	<0.001
**Age Range**	LVEF-R	323.117	2	161.558	.931	.395
	EDV-R	4778.626	2	2389.313	1.448	.236
	ESV-R	1202.309	2	601.154	.586	.557
	SD-R	12.750	2	6.375	.659	.518
	LL-R	663.551	2	331.776	1.996	.137
**Sex**	LVEF-R	8669.497	1	8669.497	49.957	<0.001
	EDV-R	151239.567	1	151239.567	91.675	<0.001
	ESV-R	62153.252	1	62153.252	60.625	<0.001
	SD-R	.017	1	.017	.002	.967
	LL-R	218.462	1	218.462	1.314	.252

**Note:** ^1^DF: degree of freedom.

**Table 7:** Effects of clinical diagnosis, age range, and sex on stress left ventricular volumetric and dyssynchrony metrics: tests of between-subjects effects.

**Source**	**Dependent Variable**	**Type III Sum of Squares**	**DF^1^**	**Mean Square**	**F**	**Sig.**
**Intercept**	LVEF-S	999999.085	1	999999.085	6854.046	<0.001
	EDV-S	3274934.140	1	3274934.140	2427.614	<0.001
	ESV-S	626467.280	1	626467.280	754.084	<0.001
	SD-S	10602.879	1	10602.879	1226.483	<0.001
	LL-S	258838.841	1	258838.841	1610.007	<0.001
**Diagnosis**	LVEF-S	17995.852	2	8997.926	61.672	<0.001
	EDV-S	133509.571	2	66754.785	49.483	<0.001
	ESV-S	102955.493	2	51477.747	61.964	<0.001
	SD-S	458.773	2	229.387	26.534	<0.001
	LL-S	5889.700	2	2944.850	18.317	<0.001
**Age Range**	LVEF-S	74.887	2	37.444	0.257	0.774
	EDV-S	9761.335	2	4880.668	3.618	0.027
	ESV-S	1161.122	2	580.561	0.699	0.497
	SD-S	30.203	2	15.102	1.747	0.175
	LL-S	211.866	2	105.933	.659	.518
**Sex**	LVEF-S	12076.612	1	12076.612	82.774	<0.001
	EDV-S	215200.924	1	215200.924	159.522	<0.001
	ESV-S	83713.267	1	83713.267	100.766	<0.001
	SD-S	65.379	1	65.379	7.563	.006
	LL-S	816.265	1	816.265	5.077	.024

**Note:** ^1^DF: degree of freedom.

**Appendix 1:** Showing age-stratified dyssynchrony variables analysis. Normal ranges are expressed as (MEAN ± SD) and (MEAN ± 2SD).

Population		**Mean Angle Mean ± SD**	**Mean Angle Mean ± 2SD**	**SD** ^◦^ **Mean ± SD**	**SD** ^◦^ **Mean ± 2SD**
ALL	S	50 (41-59)	32-68	5 (2-8)	0-11
	R	50 (42-60)	33-68	5 (2-8)	0-11
ALL (F)	S	49 (41-57)	32-65	6 (3-9)	0-11
	R	49 (41-58)	32-66	5 (3-8)	0-11
ALL (M)	S	51 (42-59)	33-68	5 (2-8)	0-12
	R	51 (43-60)	35-68	5 (2-9)	0-13
MIDDLE (F)	S	49 (40-57)	32-66	6 (3-9)	0-12
	R	49 (41-57)	32-65	6 (2-8)	0-13
SENIOR (F)	S	49 (41-57)	32-65	6 (3-9)	1-11
	R	49 (40-57)	32-66	5 (3-7)	1-11
YOUNG (F)	S	51 (42-60)	34-68	6 (3-8)	1-11
	R	54 (45-63)	36-72	6 (4-7)	2-9
MIDDLE (M)	S	51 (42-59)	34-68	5 (2-8)	0-12
	R	51 (43-59)	35-67	5 (2-9)	0-12
SENIOR (M)	S	51 (42-60)	33-68	6 (2-9)	0-13
	R	52 (44-61)	35-69	6 (2-10)	0-14
YOUNG (M)	S	50 (39-61)	29-72	5 (2-7)	0-10
	R	53 (46-60)	39-67	4 (2-7)	0-9

## Data Availability

The data of current study are available from corresponding author, [A.A], on a reasonable request. The codes are available: https://github.com/ahmaxiom/U-Net-Deep-Learning-Model-in-SPECT-Myocardial-Perfusion-Image-Segmentation.

## LIST OF ABBREVIATIONS

EDV: End-Diastolic Volume
ESV: End-Systolic Volume
LVEF: Left Ventricular Ejection Fraction
SV: Stroke Volume
ROI: Region of Interest
Mean Angle: Mean phase angle of myocardial contraction
SD°: Standard Deviation of phase angles (phase dispersion)
LL: Lower Limit of phase angle distribution
UL: Upper Limit of phase angle distribution at 25° / 75° / 95° = 25th, 75th, and 95th percentiles of the phase angle distribution
–S: Parameter measured during stress imaging
–R: Parameter measured during rest imaging
GE: General Electric (Healthcare, Chicago, IL, USA)

## STANDARDS OF REPORTING

STROBE guidelines were followed.
